# Special Hospitals

**Published:** 1892-09-03

**Authors:** 


### The Institutional Workshop.

### HOSPITAL ADMINISTRATION.

### II.—SPECIAL HOSPITALS.

In the previous article we brought out the percentage of the cost of administration as compared with that of maintenance at the principal general hospitals of the metropolis, having one hundred beds and upwards. We propose to-day to follow up our inquiries by dealing with the special hospitals on the same plan. It is very difficult to determine what constitutes a fair percentage of the cost of administration in the case of special hospitals. Most of these institutions are relatively small, and they have to maintain themselves by increased energy and continuous and extensive appeals. It has also to be remembered that there are special hospitals, and special hospitals; thus, hospitals for consumption, hospitals for children, hospitals for the paralysed, and certain opthalmic and fever hospitals, as well as the Royal Sea-bathing Infirmary, Margate, and the London Temperance, are all well entitled, from the number of beds they contain, to be classed as hospitals. When we come, however, to certain other groups which contain from twenty to sixty beds, we have to deal with institutions which are relatively so small as to be at best only cottage hospitals, and in many cases to be little better, if at all, than large out-patient departments with a few beds for certain cases, which may possess special interest or urgency, and so need in-patient treatment. Of course the question of size need have little or nothing to do with the claim of such institutions upon public support, though it has much to do with the system upon which their expenditure should be dealt with. We are of opinion that no hospital, containing less than one hundred beds, should mix the cost of the in-patients with that of the out-patients. In these cases the out-patients absorb very often two thirds or more of the whole expenditure, and so it is desirable that any outlay upon the out-patient department should be kept distinct in the accounts from that expended upon the in-patient branch of the work. We make these remarks by way of explanation, and with a view to meeting criticism like that of Mr. Kershaw, whose letter we published a few weeks ago.

We make these suggestions for a modification in the method of rendering accounts in the best interests of these relatively small hospitals, because, by its adoption, the managers will place themselves in the strongest possible position should any comparison ever be attempted between the cost of the work they do, and that which is undertaken by institutions, the bulk of whose expenditure is made upon the in-patient department. It has, however, to be borne in mind that certain diseases entail a specially heavy outlay, and with the view of arriving at a fair basis of comparison it is necessary to classify the special hospitals into groups, under their special headings, so that all those which deal with the same class of diseases may be easily compared with each other. Following out this plan, we now proceed to give a list of the metropolitan consumption hospitals:—

Metropolitan Consumption Hospitals.

| Name of Hospital. | Beds for Occupation. | Average Number Occupied. | Total In-Patients Admitted. | Percentage of Cost of Administration to that of Maintenance. |
|---|---|---|---|---|
| Brompton for Consumption ........ | 321 | 252 | 1,594 | 8·715 |
| City of London for Diseases of the Chest, Victoria Park ............ | 164 | 122 | 1,102 | 10 082 |
| Royal National, Ventnor............ | 132 | 108 | 722 | 9 267 |
| Royal for Diseases of the Chest, City Road ............ | 80 | 44 | 499 | 27 640 |
| North London, Hampstead ...... | 50 | 47 | 375 | 29·771 |

The figures in the foregoing table reveal at once the fact that in the case of a special hospital, intended for the treatment of consumption, it is desirable, for economical reasons, to have at least one hundred beds. This is apparent from the fact that whereas the three larger hospitals in the table expend less together than 10 per cent. upon administration as compared with maintenance, the two smaller institutions, which contain eighty and fifty beds respectively, of which forty-four and forty-seven beds respectively were occupied throughout the year, cost in each case for administration more than three times as much as the Hospital for Consumption, Brompton. We commend these figures to the consideration of the committees and the public. We would remind both that in the case of the North London, Hampstead, at any rate, there is a splendid site not yet occupied by buildings, and that London at the present time urgently needs many hundreds more hospital beds for the reception of cases of phthisis. There are in the metropolis, and throughout the country—and the question of providing an adequate number of beds for consumptive cases is a country question quite as much, if not more, than it is a metropolitan one, because so many of the patients in the consumption hospitals are at present sent from the country—an increasing number of wealthy people who desire to give of their abundance substantial sums for the alleviation of suffering in their day and generation. We know no better object upon which they can expend a very large sum of money than by placing themselves in communication with the Chairman of the North London Consumption Hospital, Hampstead, or with the Chairman of the Royal Hospital for Diseases of the Chest, City Road—though in the latter case the site, we believe, is fully occupied—with the object of concerting measures to considerably extend the number of beds at present available in these institutions for the reception and treatment of urgent cases of consumption.

Consumption may be regarded as the curse of our climate; it spares neither prince nor peasant; it is met with in the homes of the wealthy, and in the cottages of the poor, and so all classes, and each class for itself, is personally interested in securing an adequate number of hospital beds in our consumption hospitals, so that science may have the fullest opportunity of discovering the best methods of treatment, and each of us may do our utmost to alleviate a form of human misery which appeals, by the very nature of its insidious but fatal progress, to the best instincts of every healthy man and woman in our midst. Would that our words could reach the ears, and that they might quicken the sense and understanding of those wealthy people who have more money than they know what to do with. One way of securing this result would be for the authorities of the consumption hospitals to take care that the facts here brought out, and the appeal now made, shall be sent to the right quarter, and then we make no doubt that the response will be adequate. If so, it will come to pass that the hospital provision for consumption will soon bear a better proportion to the needs and necessities of thousands of poor sufferers who at present languish and suffer—we had almost said unnecessary—pain and misery, because the public conscience has not yet been awakened to the urgent and pressing claims which both ought to have upon the pocket, the heart, and the conscience of all well-to-do people.

